# Perception de la carence martiale et de l'anémie ferriprive par les médecins de différentes spécialités en Algérie en 2016: enquête SUPFER DZ

**DOI:** 10.11604/pamj.2019.33.48.15114

**Published:** 2019-05-22

**Authors:** Rosa Belkaid, Malek Benakli, Naima Hammoudi-Bendib, Nadjia Ramdani-Bouguessa, Lamine Mahi

**Affiliations:** 1 Service d'Epidémiologie et de Médecine Préventive, Centre Hospitalier Universitaire Béni-Messous, Alger, Algérie; 2 Service d'Hématologie-Greffe de Moelle Osseuse, Centre Pierre et Marie Curie, Alger, Algérie; 3 Service de Cardiologie, Etablissement Hospitalier Spécialisé, Docteur Maouche Mohand Amokrane, Alger, Algérie; 4 Laboratoire de Biologie, Alger, Algérie; 5 Axelys Santé DZ, Alger, Algérie

**Keywords:** Carence martiale, anémie ferriprive, fer oral, fer injectable, Iron deficiency, iron deficiency anemia, oral iron, iron injection

## Abstract

**Introduction:**

Les méthodes de diagnostic et la prise en charge de la carence martiale et de l'anémie ferriprive en pratique clinique en Algérie restent mal connues.

**Méthodes:**

Enquête transversale réalisée en 2016 auprès de médecins de différentes spécialités concernés par la prise en charge de l'anémie ferriprive.

**Résultats:**

Pour l'analyse des données, 349 questionnaires ont été validés (anesthésie/réanimation: 39; gynécologie/obstétrique: 111; oncologie/hématologie: 71; hépato-gastroentérologie: 64; cardiologie: 36; médecine interne: 28). Toutes spécialités confondues, 73% (254/349) des médecins estimaient que l'anémie ferriprive concernait au moins 30% de leur patientèle; 65% (226/349) estimaient que la carence martiale concernait au moins 30% de leur patientèle. La carence martiale était explorée systématiquement par 57% (63/111) des médecins du groupe gynécologie/obstétrique, mais par 11% (26/238) seulement des autres médecins; en effet, 82% (195/238) ne la recherchaient qu'en cas d'anémie. Dans le cadre d'un bilan de carence martiale, l'hémoglobine (Hb) était presque toujours évaluée (89%; 310/349) alors que les examens biologiques qui ciblent directement le métabolisme du fer l'étaient insuffisamment: par 70% (244/349) des médecins pour la ferritine sérique et par seulement 37% (128/349) pour le coefficient de saturation de la transferrine. En cas de carence martiale (avec ou sans anémie), le fer oral était prescrit par 92% (322/349) des médecins et 36% (127/349) prescrivaient du fer injectable en tenant compte essentiellement du taux d'Hb.

**Conclusion:**

Cette enquête montre que la carence martiale semble n'être envisagée que dans le cadre de l'anémie. En particulier, les examens biologiques spécifiques de la carence martiale sont insuffisamment prescrits.

## Abstract (en)

**Introduction:**

diagnostic methods and management of iron deficiency and iron deficiency anemia in clinical practice in Algeria is poorly known.

**Methods:**

we conducted a cross-sectional survey among doctors in different specialties treating patients with iron deficiency anemia in 2016.

**Results:**

data analysis was based on 349 questionnaires which were validated (anesthesia/resuscitation: 39; obstetrics and gynaecology: 111; oncology/Hematology: 71; hepato-gastroenterology: 64; cardiology: 36; internal medicine: 28). All specialties combined, 73% (254/349) of physicians thought that at least 30% of their patients had iron deficiency anemia; 65% of physicians (226/349) thought that at least 30% of their patients had iron deficiency. Iron deficiency was investigated systematically by 57% (63/111) of physicians of the group obstetrics and gynaecology, but only by 11% (26/238) of the remaining doctors; indeed, 82% (195/238) of physicians investigated it only in patients with anemia. The assessment of iron deficiency showed that the hemoglobin (Hb) was almost always determined (89%; 310/349) while laboratory tests to explore iron metabolism were inadequate: 70% (244/349) of physicians performed serum ferritin test and only 37% (128/349) performed transferrin saturation. Patients with iron deficiency (with or without anemia) received oral iron therapy (prescribed by 92% (322/349) of physicians) and iron injections therapy depending on Hb level (prescribed by 36% (127/349) of physicians).

**Conclusion:**

this survey shows that iron deficiency is evaluated only in patients with anemia. In particular, laboratory tests to measure iron deficiency are insufficiently prescribed.

## Introduction

Selon l'Organisation Mondiale de la Santé, la carence en fer est le trouble nutritionnel le plus fréquent dans le monde; on estime que deux milliards de personnes souffrent d'anémie, principalement en raison d'une carence en fer [1]. Cette carence est aggravée par les maladies infectieuses et inflammatoires [2]. Le renouvellement quotidien des hématies nécessite en effet des quantités importantes de fer. L'anémie ferriprive ne survient toutefois qu'à un stade tardif de la carence martiale. En dehors du transport de l'oxygène, le fer joue également un rôle important dans de nombreux processus enzymatiques ou métaboliques (respiration cellulaire, détoxication, protection contre le stress oxydatif ou métabolisme énergétique) [3]. C'est pourquoi une carence martiale, en dehors de toute anémie, peut être responsable de symptômes divers tels que fatigue, diminution des performances physiques, troubles cognitifs et perturbations de la thermorégulation [4, 5]. Il est important de souligner qu'une supplémentation en fer permet d'améliorer ces symptômes, y compris en dehors de toute anémie [6-9]. Par ailleurs les connaissances sur le métabolisme du fer ont été considérablement enrichies au cours des dernières années avec la découverte de l'hepcidine, une protéine qui régule le transport du fer [10, 11]. De plus, de nouveaux complexes de fer injectable ont été mis à la disposition des médecins. Les formes injectables sont souvent plus efficaces que le fer oral, en particulier chez les patients ayant des maladies chroniques ou inflammatoires [12]. Les nouvelles données fondamentales sur le métabolisme du fer et la prévalence importante de la carence martiale devraient inciter à dépister et à supplémenter les sujets carencés. Les pratiques des médecins restent toutefois mal connues et de nombreux patients pourraient certainement bénéficier d'une prise en charge plus précoce. Le but de cette étude était d'évaluer la perception, les méthodes de diagnostic et la prise en charge de la carence martiale et de l'anémie ferriprive en pratique clinique en Algérie.

## Méthodes

Il s'agissait d'une enquête qualitative transversale. Les centres importants en termes de représentativité et de répartition géographique ont été identifiés à partir d'un annuaire professionnel. Ils constituent un réseau sentinelle en matière d'offres et d'accès aux soins. Les médecins qui ont accepté de participer à l'enquête ont reçu des questionnaires standardisés auto-administrés qui comportaient des questions communes à l'ensemble des spécialités et des questions plus spécifiques pour certains groupes de spécialités (gynécologie/obstétrique, oncologie/hématologie, anesthésie/réanimation/chirurgie). Les questions portaient sur les fréquences estimées de l'anémie et de la carence martiale parmi les patients suivis par le médecin, les raisons de la recherche d'une carence martiale, les examens utilisés pour le diagnostic, le traitement de la carence martiale, les conditions d'utilisation du fer injectable, l'utilisation éventuelle d'agents stimulant l'érythropoïèse dans le traitement de l'anémie en association ou non avec une supplémentation en fer, les critères de prescription de fer injectable et les critères d'arrêt du traitement d'une carence martiale. Pour l'analyse, les médecins ont été regroupés selon leurs spécialités médicales: anesthésie/réanimation, chirurgie, gynécologie/obstétrique oncologie/hématologie, hépato-gastroentérologie, cardiologie, médecine interne, autres spécialités.

## Résultats

**Caractéristiques des médecins de l'enquête**: parmi les 400 médecins contactés pour cette étude, 38 (9,5%) n'ont pas souhaité participer. Au total, les réponses de 362 médecins de différentes spécialités ont été analysées (anesthésie/réanimation, n=39; chirurgie, n=4; gynécologie/obstétrique, n=111; oncologie/hématologie, n=71; hépato-gastroentérologie, n=64; cardiologie, n=36; médecine interne, n=28; autres spécialités, n=9). Pour la suite de l'analyse, les réponses des médecins des groupes chirurgie et autres spécialités n'ont pas été prises en compte du fait des faibles effectifs. Au total, les réponses de 349 médecins ont été analysées. Les médecins de l'enquête étaient des femmes pour 72% (252/349) et ils étaient âgés en moyenne de 33 ans (min-max, 25-60 ans) (Tableau 1); les plus jeunes étaient les médecins du groupe gynécologie/obstétrique (30 ans), ce groupe étant également celui où la proportion de femmes était la plus élevée (92%; 102/111). Les deux tiers des médecins avaient un exercice urbain (66%; 231/349) (Tableau 1).

**Tableau 1:** Âge, sexe et exercice rural/urbain des 349 médecins de l’étude selon les spécialités

	Onco-Hématologie (n = 71)	Hépato-Gastro- Entérologie (n = 64)	Médecine interne (n = 28)	Cardiologie (n = 36)	Gynécologie/ Obstétrique (n = 111)	Anesthésie/Réanimation (n = 39)
Sexe féminin, n (%)	46(65)	39(61)	24(86)	14(39)	102(92)	27(69)
Age moyen en années (SD)	33(6)	31(6)	35(12)	32(8)	30(6)	36(8)
**Mode d’exercice, n (%)**						
Rural	39(55)	10(16)	15(54)	5(14)	30(27)	19(49)
Urbain	32(45)	54(84)	13(46)	31(86)	81(73)	20(51)

**Figure 1:**
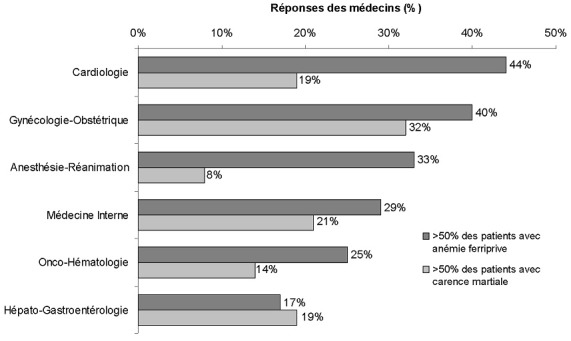
Fréquence de la carence martiale et de l'anémie ferriprive estimée par les médecins de l'enquête parmi leurs patients

**Fréquences de la carence martiale et de l'anémie ferriprive selon les déclarations des médecins**: la carence martiale et l'anémie sont des situations cliniques souvent rencontrées par les médecins de l'enquête: toutes spécialités confondues, 73% des médecins estimaient que l'anémie ferriprive concernait au moins 30% de leur patientèle; 65% des médecins estimaient que la carence martiale concernait au moins 30% de leur patientèle (Tableau 2). Les fréquences rapportées étaient toutefois hétérogènes selon les spécialités (Figure 1). Des fréquences >50% de l'anémie ferriprive parmi les patients suivis étaient rapportées plus fréquemment dans les groupes cardiologie et gynécologie-obstétrique (respectivement 44% et 40% des réponses des médecins). Dans le groupe gynécologie/obstétrique, 32% des médecins estimaient que la fréquence de la carence martiale parmi leurs patients était >50% (Figure 1).

**Tableau 2:** Fréquence de la carence martiale et de l’anémie ferriprive selon les médecins de l’enquête parmi leurs patients et utilisation de fer injectable pour une carence martiale

Questions de l’enquête	N=349	Questions de l’enquête	N=349
**Fréquence de l’anémie ferriprive**		**Traitement d’une carence martiale (avec ou sans anémie) ^a^**	
<10%	17(5%)	Apport de fer par voie orale	322(92%)
10 à 20%	25(7%)	Utilisation de fer injectable	127(36%)
20 à 30%	47(13%)	Transfusion	168(48%)
30 à 40%	53(15%)	Pas de réponse	31(9%)
40 à 50%	89(26%)		
> 50%	110(32%)	**Critères d’utilisation de fer injectable en première intention ^a^**	
Pas de réponse	8(2%)	CST	21(6%)
**Fréquence de la carence martiale**		Ferritinémie	50(14%)
<10%	19(5%)	Taux d’Hb, g/dL	195(56%)
10 à 20%	40(11%)		
20 à 30%	49(14%)	Utilisation d’ASE dans le traitement de l’anémie	N=238
30 à 40%	71(20%)	**(hors Gynécologie/Obstétrique)**	
40 à 50%	81(23%)	Utilisation occasionnelle	91(38%)
> 50%	74(21%)	**Supplémentation en fer avec ASE**	
Pas de réponse	15(4%)	Non	27/91(30%)
**Recherche d’une carence martiale**		Fer oral	45/91(49%)
Systématiquement	89(26%)	Fer injectable	18/91(20%)
Uniquement si anémie	241(69%)	Pas d’utilisation d’ASE	106(46%)
Non	17(5%)	Pas de réponse	41(17%)
Pas de réponse	2(1%)		

a Plusieurs réponses possibles. ASE: agent stimulant l’érythropoïèse ; CST: coefficient de saturation en fer de la transferrine

**Figure 2:**
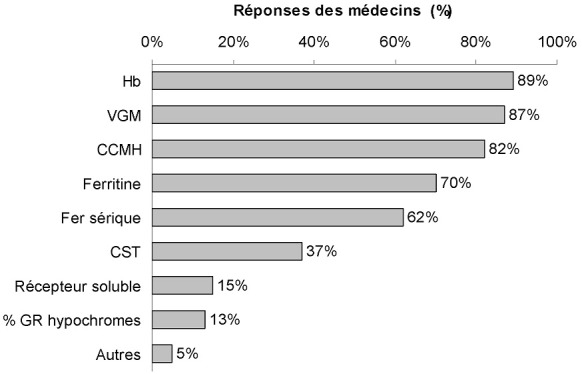
Examens biologiques prescrits par les médecins de l'enquête pour le diagnostic de carence martiale

**Figure 3:**
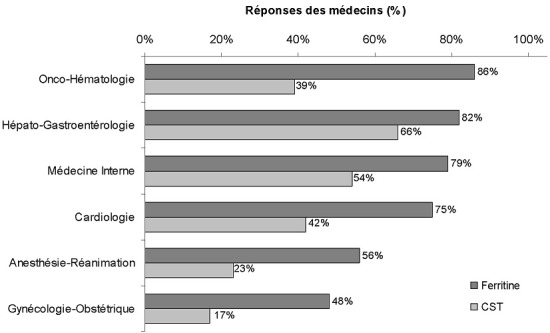
Mesure de la ferritine sérique et du CST dans le cadre d'un diagnostic de carence martiale en fonction des spécialités des médecins de l'enquête

**Recherche de la carence martiale et examens biologiques pour son diagnostic**: globalement, 26% (89/349) des médecins recherchaient systématiquement une carence martiale (Tableau 2). Cette recherche systématique était toutefois très hétérogène selon les spécialités. Ainsi, en dehors du groupe gynécologie/obstétrique, seulement 11% (26/238) des médecins de l'enquête recherchaient systématiquement une carence martiale et 82% (195/238) la recherchaient uniquement en cas d'anémie. En revanche, les médecins du groupe gynécologie/obstétrique recherchaient la carence martiale de manière systématique plus souvent que les médecins des autres spécialités (57%; 63/111). En particulier, 62% (69/111) des médecins du groupe gynécologie/obstétrique recherchaient la carence martiale systématiquement en cas de ménométrorragies (23% uniquement en cas d'anémie et 14% uniquement en cas d'anémie microcytaire). Parmi les médecins du groupe anesthésie/réanimation, l'anémie ferriprive était recherchée systématiquement en préopératoire (hors urgence) par 62% (24/399) d'entre eux et selon les pertes sanguines prévisibles par 15% (6/39), en général deux semaines avant la date prévue pour l'intervention chirurgicale. Ce sont surtout les examens biologiques qui correspondent aux conséquences tardives de la carence martiale (c'est-à-dire l'anémie ferriprive) qui étaient pris en compte par les 349 médecins de l'enquête afin d'établir le diagnostic de carence martiale: Hb (89%; 310/349), volume globulaire moyen (VGM) (87%; 305/349) et concentration corpusculaire moyenne en hémoglobine (CCMH) (82%; 285/349) (Figure 2). En revanche, les paramètres biologiques qui explorent plus directement le métabolisme du fer étaient utilisés beaucoup moins fréquemment. Ainsi la ferritine sérique et le coefficient de saturation de la transferrine (CST) étaient prescrits respectivement par seulement 70% (244/349) et 37% (128/349) des médecins pour le diagnostic de carence martiale. À noter que le fer sérique était prescrit par près des deux tiers (62%; 215/349) des médecins. Une analyse plus fine des réponses des médecins montre que la mesure de la ferritine sérique et du coefficient de saturation de la transferrine (CST) variait beaucoup selon les spécialités médicales: respectivement 82% (56/64) et 66% (42/64) pour le groupe hépato-gastroentérologie, mais seulement 48% (53/111) et 17% (19/111) pour le groupe gynécologie/obstétrique (Figure 3).

**Figure 4:**
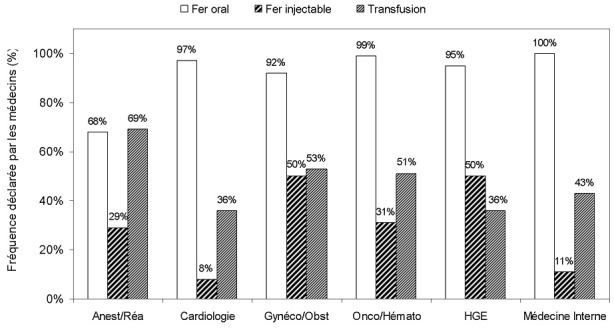
Traitement de la carence martiale (avec ou sans anémie)

**Traitement de la carence martiale**: pour une large majorité de médecins, le traitement de la carence martiale (avec ou sans anémie) reposait avant tout sur le fer oral (92%; 322/349) (Tableau 2). L'utilisation du fer injectable était rapportée par 36% des médecins (127/349) mais de façon variable selon les spécialités: seulement 8% (3/36) en cardiologie et 11% (3/28) en médecine interne mais 50% (56/111) en gynécologie/obstétrique et 50% (32/64) en hépato-gastroentérologie (Figure 4). Le recours à la transfusion était mentionné par 48% (168/349) des médecins. Les médecins du groupe anesthésie/réanimation avaient recours au fer injectable en préopératoire pour 69% (27/39) ou en post-opératoire pour 64% (25/39). Dans le groupe gynécologie/obstétrique, le traitement de première intention de l'anémie ferriprive était majoritairement réalisé par le fer oral au cours du 1^er^ trimestre (89% vs. 11% pour le fer injectable), du 2^ème^ trimestre (81% vs. 15%) et du post partum (64% vs. 43%); pour le 3^ème^ trimestre, le fer injectable était préféré par la majorité des médecins (62% vs. 45%). En cas de carence martiale, c'est majoritairement le taux d'Hb (< 8 g/dL en médiane sauf pour le groupe gynécologie/obstétrique: < 7 g/dL) qui conduisait les médecins à administrer du fer sous forme injectable (56%; 195/349); les résultats de la ferritinémie et du CST intervenaient dans cette décision respectivement chez 14% (50/349) et 6% (21/349) des médecins seulement. Le traitement de la carence martiale était arrêté après correction de l'anémie pour 28% (99/349) des médecins, la correction du bilan martial pour 42% (145/349), une dose totale calculée pour 7% (24/349) ou une durée fixe pour 22% (76/349).

**Utilisation des agents stimulants de l'érythropoïèse dans l'anémie**: parmi l'ensemble des médecins (hors groupe gynécologie/obstétrique), 31% (91/349) rapportaient qu'ils utilisaient occasionnellement des agents stimulants de l'érythropoïèse (ASE) dans le traitement de l'anémie. L'usage des ASE était rapporté surtout par les médecins des groupes oncologie-hématologie (75%; 53/71), hépato-gastroentérologie (28%; 18/64) et médecine interne (32%; 9/28). Parmi ces utilisateurs occasionnels d'ASE, 30% (27/91) n'associaient jamais une supplémentation en fer et, lorsque c'était le cas, c'était plus souvent du fer oral (49%; 45/91) que du fer injectable (20%; 18/91).

## Discussion

L'un des principaux enseignements de cette enquête est que la carence martiale est considérée par les médecins essentiellement sous l'angle de l'anémie ferriprive. Ainsi, parmi les tests biologiques prescrits dans le cadre du diagnostic de carence martiale, ce sont les paramètres de l'hémogramme (Hb, VGM, CCMH) qui étaient largement pris en considération alors que les évaluations directes des réserves en fer et de son transport (ferritine sérique et CST) étaient sous-représentées. L'anémie ferriprive est en effet un stade tardif de la carence martiale. Au stade initial, les réserves en fer diminuent sans provoquer d'anomalies de l'hémogramme. La ferritine sérique est alors le paramètre le plus fiable pour évaluer les réserves tissulaires en fer [13]. C'est seulement dans un deuxième temps qu'une diminution de la CCMH et du VGM (microcytose) est observée [14]. Enfin, si la carence martiale s'accentue, l'anémie apparaît (définie par Hb <12 g/dL chez les femmes non enceintes et <13 g/dL chez les hommes) [15]. L'Hb et les autres paramètres de l'hémogramme sont donc des marqueurs peu sensibles de la carence martiale. On remarque que l'évaluation de la ferritine sérique pour le diagnostic de la carence martiale variait grandement selon la spécialité: elle était plus fréquemment rapportée dans les groupes onco-hématologie, hépato-gastroentérologie et médecine interne par au moins 4 médecins sur 5. Il convient de noter à ce propos qu'une majorité de médecins de l'enquête continuait de mesurer le fer sérique (64%) alors qu'il est maintenant établi que cet examen n'est pas fiable du fait de ses variations au cours du nycthémère. La mesure du CST était rapportée surtout par les médecins des groupes hépato-gastroentérologie et médecine interne mais à des taux qui pourraient être améliorés.

La ferritine sérique est le marqueur de référence de la carence martiale et elle doit être prescrite en première intention dans le cadre d'une exploration du statut martial. Si la ferritine sérique est le reflet du fer stocké dans les réserves, le CST quant à lui est le reflet du fer de transport. Lorsque fer de réserve et fer de transport sont diminués (ferritine sérique < 30 µg/L et CST < 20%), on parle de carence martiale absolue. Cette situation est observée en cas d'insuffisance d'apport de fer ou de pertes sanguines. La carence martiale est dite fonctionnelle lorsque les réserves de fer ne sont pas affectées (ferritine sérique normale ou augmentée) mais que sa mobilisation vers les lieux de son utilisation est perturbée (CST < 20%). Cette situation est rencontrée dans les états inflammatoires chroniques. On parle alors de « séquestration » tissulaire du fer [2]. Avec les pertes menstruelles et la grossesse qui augmentent les besoins en fer, les femmes en âge de procréer sont une population cible pour la prévention de la carence martiale. Moulessehoul *et al.* ont étudié dans un centre de protection infantile à Sidi Bel Abbès l'impact de la supplémentation en fer de femmes enceintes [16]. Au cours du premier trimestre de la grossesse, 60% des 83 femmes de l'étude présentaient une carence martiale (évaluée par le fer sérique) et 31% une anémie (Hb < 11 g/dL). Une autre étude réalisée à Blida a montré également la prévalence importante de l'anémie ferriprive chez des femmes enceintes (47% à la fin de la grossesse) [17]. Ces résultats sont à rapprocher de ceux de notre enquête. En effet, les médecins gynécologues-obstétriciens semblent les plus sensibilisés à la question du statut martial puisqu'ils déclaraient majoritairement (contrairement aux autres spécialistes) qu'ils réalisaient systématiquement une exploration du statut martial. Logiquement ce sont eux qui déclaraient également le plus de carences martiales (un tiers de leurs patientes). Mais, paradoxalement, ils étaient les moins nombreux (la moitié environ) à mesurer la ferritine sérique pour étayer le diagnostic de la carence martiale.

Le traitement de la carence martiale était principalement le fer oral pour la quasi-totalité des médecins de l'étude. L'utilisation du fer injectable semble réservée aux cas d'anémies sévères puisque c'est la valeur du taux d'Hb (< 8 g; sauf pour le groupe gynécologie/obstétrique: < 7 g/dL) qui guide principalement sa prescription par les médecins. Le fer injectable peut avoir certains avantages chez les patients avec une anémie ferriprive sévère, les patients hémodialysés, ceux qui tolèrent mal le fer oral ou en cas d'inefficacité du fer oral. Le fer injectable a également l'avantage sur le fer oral de court-circuiter la barrière intestinale. En cas d'inflammation chronique, fréquente au cours des maladies chroniques, il est maintenant bien établi que le fer oral est généralement inefficace. Son absorption intestinale est en effet bloquée, en parallèle avec la « séquestration » du fer tissulaire [2]. Plusieurs études cliniques randomisées ont montré la supériorité du fer injectable comparé au fer oral au cours de l'anémie ferriprive du post-partum [18-20]. Parmi les médecins qui utilisaient occasionnellement des ASE, 30% n'associaient jamais une supplémentation en fer et parmi ceux qui le faisaient c'était le plus souvent avec du fer oral. Or, la stimulation de l'érythropoïèse mobilise des quantités importantes de fer à partir des réserves. Il est maintenant bien établi que la supplémentation en fer permet non seulement d'augmenter la réponse au traitement mais également de diminuer les doses d'ASE [21, 22].

## Conclusion

Cette étude qualitative met en évidence certains points intéressants quant à la perception de la carence martiale par les médecins algériens. Ainsi, la carence martiale semble se confondre avec l'anémie ferriprive dans l'approche diagnostique des médecins de l'étude et par conséquent les tests spécifiques de la carence martiale (ferritine sérique en particulier) sont sous-utilisés. Cette méconnaissance du métabolisme du fer et des traitements qui permettent de prévenir et traiter la carence martiale indiquent que des efforts pédagogiques pourraient contribuer efficacement à la lutte contre la carence martiale. De même un consensus pour le diagnostic et le traitement de la carence martiale devrait être établi par un groupe d'experts pluridisciplinaire.

### Etat des connaissances actuelles sur le sujet

- La carence en fer est le trouble nutritionnel le plus fréquent dans le monde;
- Les pratiques des médecins concernant le diagnostic et la prise en charge de la carence en fer restent mal connues;
- De nombreux patients pourraient bénéficier d'une prise en charge plus précoce avant la survenue d'une anémie ferriprive.

### Contribution de notre étude à la connaissance

- Il existe de grandes disparités concernant le diagnostic et la prise en charge de la carence en fer selon les spécialités;
- Pour une majorité de médecins, la carence en fer n'est envisagée que dans le cadre de l'anémie ferriprive;
- Les examens biologiques qui ciblent directement le métabolisme du fer (ferritine et coefficient de saturation de la transferrine) sont insuffisamment prescrits.
